# Dietary Fiber Levels Modulate Intestinal Mucosal Architecture and the Microbiome–Metabolome Axis to Support Immune Homeostasis in Brooding Wanxi White Geese

**DOI:** 10.3390/ani16111709

**Published:** 2026-06-03

**Authors:** Zhiying Yuan, Fei Xie, Yuancui Ding, Xiaojin Li, Ahmed H. Ghonaim, Changsheng Jiang, Man Ren, Shenghe Li

**Affiliations:** 1 College of Animal Science, Anhui Science and Technology University, Chuzhou 233100, China; 13142153459@163.com (Z.Y.); xiefei19980430@126.com (F.X.); yuancuiding@163.com (Y.D.); lixj@ahstu.edu.cn (X.L.); jiangcs@ahstu.edu.cn (C.J.); 2 Anhui Province Key Laboratory of Animal Nutritional Regulation and Health, Chuzhou 233100, China; 3 Local Geese Gene Bank in Anhui Province, Chuzhou 233100, China; 4 National Key Laboratory of Agricultural Microbiology, College of Veterinary Medicine, Huazhong Agricultural University, Wuhan 430070, China; drcahmed91@gmail.com; 5 Desert Research Center, Cairo 11753, Egypt

**Keywords:** Wanxi White Goose, dietary fiber, gut morphology, gut microbiota, metabolomics

## Abstract

Dietary fiber is a critical determinant of intestinal health, yet its optimal inclusion level for WWG during the critical brooding period remains undefined. This study aimed to evaluate the effects of varying dietary CF levels (approximately 3%, 5%, and 9%) on the intestinal morphology, immune function, and microbiome-metabolome axis of brooding WWG. A total of 120 one-day-old goslings were randomly assigned to the three dietary treatments for a 28-day trial. Histological analysis revealed that the 9% CF diet significantly improved gut morphology, yielding superior villus-to-crypt ratios in the jejunum and ileum. Molecular assays indicated that higher fiber levels (5–9%) upregulated the expression of nutrient transporters (SGLT1 and GLUT2). Concurrently, the 9% CF diet effectively suppressed the potent pro-inflammatory cytokine TNF-α in the jejunum while appropriately upregulating IL-6 and NF-κB, indicating enhanced mucosal immune vigilance and structural maturation. Multi-omics integration (shotgun metagenomics and LC-MS metabolomics) demonstrated that specific fiber levels significantly shifted microbial abundances, specifically enriching Bacteroidetes and Actinobacteria. These microbial shifts were strongly correlated with enriched metabolic pathways, notably lysine biosynthesis and purine metabolism, which synergistically support mucosal homeostasis. Collectively, these findings demonstrate that a 9% dietary CF inclusion is an effective nutritional strategy to optimize intestinal architecture and microbial-metabolic profiles in brooding WWG.

## Simple Summary

Identifying the optimal dietary fiber level is crucial for the sustainable and cost-effective farming of Wanxi White Geese (WWG). While fiber is essential for poultry gut health, insufficient or excessive amounts can limit nutrient utilization during the critical early brooding stage. This study reveals that feeding brooding WWG a diet formulated with 9% crude fiber (CF) significantly fortifies their intestinal physical structure, promotes a beneficial gut microbial balance, and supports robust early immune defense. For poultry producers, implementing this 9% fiber feeding strategy offers a highly practical, science-based approach to enhance early gut development, improve feed efficiency, and ultimately increase the overall health and economic viability of goose farming operations.

## 1. Introduction

China is a global leader in goose production and consumption, accounting for more than 90% of the world’s goose population. As a result, the goose industry plays a significant role in supporting the national economy. Geese are predominantly herbivorous poultry with strong tolerance for high-fiber diets, low feed intake, limited input requirements, and high economic returns. Their ability to efficiently utilize forages and crop residues not only conserves grain resources but also helps mitigate pressures associated with China’s expanding population and declining arable land. Thus, advancing research on their nutritional requirements is essential for reducing feed costs and promoting sustainable industry development. However, studies on the digestive physiology and nutrient requirements of geese remain insufficient, particularly regarding optimal dietary fiber levels across developmental stages [1,2].

In poultry, intestinal health is closely associated with production performance and product safety in poultry. Dietary fiber influences intestinal function by modulating gut development, mucosal morphology, and microbial ecology, thereby improving feed utilization efficiency and growth performance [3,4]. Evidence indicates that moderate fiber supplementation promotes gastrointestinal development in poultry and enhances nutrient absorption, productivity, and disease resistance in poultry [5,6]. With the rapid progress in high-throughput sequencing technologies, research on the structure and function of the gut microbiota, and its multifaceted effects on the host, has become increasingly prominent. The gut microbiota is now recognized as a key determinant of poultry health and metabolic regulation [7,8]. Furthermore, the gut microbiota exerts its profound effects on host health primarily through a complex network of metabolic outputs. Microbial metabolites, including specific organic acids and synthesized essential amino acids, act as crucial signaling molecules that directly modulate intestinal structural integrity and local immune homeostasis. Despite this critical host–microbe metabolic crosstalk, how specific dietary fiber levels drive these metabolomic shifts to fortify the intestinal immune barrier in geese remains largely unexplored.

Geese possess a distinct digestive physiology that supports efficient fiber utilization, including a spacious and flexible esophagus, a highly muscular gizzard with strong grinding capacity, and an acidic gastrointestinal environment conducive to fiber degradation [9,10]. Microbial richness and diversity in geese increase along the gastrointestinal tract, reaching their peak in the ceca, which harbors a dense and metabolically active microbiota. Consequently, the cecum has become the most frequently examined intestinal segment in poultry microbiome studies. Moreover, the goose gut microbiota undergoes a developmental succession from a relatively simple, transient community in early life to a more stable and diverse ecosystem as the birds mature [11,12]. Despite this importance, research on goose gut microbiota remains scarce compared with that in chickens and ducks.

The brooding period is a critical developmental stage for the maturation of the gastrointestinal tract and immune system in geese. In this study, Wanxi White Geese (WWG) during the brooding period were used as the experimental model to investigate the effects of different dietary crude fiber (CF) levels on intestinal morphology, immune function, and microbial ecology. Using integrated metagenomic and untargeted metabolomic analysis, we aimed to decipher the specific microbiome–metabolome axis regulated by dietary fiber. We systematically evaluated how optimal fiber levels reshape microbial metabolic profiles, particularly those related to amino acid and lipid metabolism, to maintain mucosal immune homeostasis. Ultimately, this study seeks to evaluate different dietary fiber levels to identify an effective feeding strategy that fully unlocks the early growth potential of brooding geese and improves their intestinal health, providing a robust theoretical foundation for the rational application of fibrous feed ingredients in WWG production.

## 2. Materials and Methods

### 2.1. Ethics Statement

All animal experimental protocols were conducted in accordance with the guidelines for the care and use of experimental animals established by the Institutional Animal Care and Use Committee of the Anhui Science and Technology University, Fengyang, China. Ethical approval was granted by the same committee (Approval No. 2023-014).

### 2.2. Experimental Design and Feeding Management

A total of 120 healthy one-day-old WWG were obtained from the Junming Goose Breeding Base (Dingyuan County, China). Goslings were randomly assigned to three dietary treatments formulated to contain different levels of CF (3.52%, 5.07%, and 9.00%, designated hereafter as the 3%, 5%, and 9% fiber groups, respectively). These CF levels correspond to total dietary fiber contents of 16.07%, 18.33%, and 24.33%. The experimental period lasted 28 days. All birds were raised on elevated mesh flooring with ad libitum access to feed and water. Routine management and health-care procedures followed standard production practices.

### 2.3. Diet Composition and Nutrient Levels

Experimental diets were formulated using corn and soybean meal as basal ingredients, with rice hull meals as the fiber source. Nutrient contents were calculated according to the China Feed Composition and Nutritional Value Table (2021 edition). The detailed formulation and nutritional composition of the basal diets are presented in Table 1.

### 2.4. Sample Collection

At 28 days of age, five healthy geese with comparable body weights were randomly selected from each group and euthanized for sampling. For all subsequent histological, targeted molecular, and untargeted omics evaluations, the individual bird was considered the experimental unit. Segments of the duodenum, jejunum, ileum, and cecum, along with corresponding digesta, were collected. Portions of each intestinal segment were fixed in preservative solution for morphological examination. The remaining tissue and digesta samples were snap-frozen in liquid nitrogen and stored at −80 °C for sequencing analyses. Metagenomic and metabolomic sequencing was performed to Shanghai Majorbio Bio-pharm Technology Co., Ltd. (Shanghai, China).

### 2.5. Small Intestine Tissue Sectioning and Observation

Intestinal segments (duodenum, jejunum, and ileum) were systematically sampled from the mid-point of each respective section. The samples, approximately 1 cm in length, were immediately fixed in 4% paraformaldehyde solution at a tissue-to-fixative volume ratio of at least 1:10 for 24 to 48 h. Fixed tissues were trimmed, dehydrated through a graded ethanol series, cleared in xylene, and transversely embedded in paraffin. Sections (5 µm) were prepared and stained with Hematoxylin and Eosin (H&E) (Solarbio, Beijing, China). Slides were scanned using an Olympus BX63 (Olympus, Tokyo, Japan) upright fluorescence microscope. Histomorphological analyses were conducted using Motic DSAssistant Lite (Motic, Xiamen, China), and villus height (VH) and crypt depth (CD) were measured with Image-Pro Plus 6.0 software to calculate the villus height-to-crypt depth (V/C) ratio. For quantitative accuracy, 3 to 5 non-consecutive sections per intestinal sample were evaluated. Within each section, a minimum of 10 intact, well-oriented villi and their associated crypts were measured. Strict exclusion criteria were applied: only villi with a continuous epithelial layer and visible lamina propria from the tip to the crypt base were selected, while oblique, heavily folded, or mechanically damaged sections were excluded. To eliminate subjective bias and ensure statistical robustness, all measurements were performed by a single trained investigator who was strictly blinded to the dietary treatment groups, which effectively minimized intra-observer variability.

### 2.6. Assessment of Intestinal Immune Function

The concentrations of secretory immunoglobulin A (sIgA), interleukin-6 (IL-6), interleukin-10 (IL-10), interferon-gamma (IFN-γ), nuclear factor-kappa B (NF-κB), and tumor necrosis factor-alpha (TNF-α) in the jejunum and ileum were quantified using commercial enzyme-linked immunosorbent assay (ELISA) kits (Shanghai Xinle Biotechnology Co., Ltd., Shanghai, China) according to the manufacturer’s protocols. Briefly, frozen intestinal tissue samples were homogenized in ice-cold phosphate-buffered saline at a weight-to-volume ratio of 1:9. The homogenates were subsequently centrifuged at 3000× *g* for 15 min at 4 °C. The resulting supernatants were carefully collected and added to pre-coated microplates, followed by incubation with horseradish peroxidase-conjugated antibodies. After thorough washing to remove unbound components, substrate solutions were added for color development, and the reaction was terminated using a stop solution. The optical density of each well was measured at 450 nm using a microplate reader (Tecan, Zürich, Switzerland). Final concentrations were calculated based on the standard curves and normalized to the total tissue protein content.

### 2.7. Intestinal Gene Expression

Total RNA was extracted from the tissues of the duodenum, jejunum, and ileum using Trizol reagent (Invitrogen, Carlsbad, CA, USA). RNA integrity was verified by agarose gel electrophoresis, and purity was assessed using a Nanodrop One spectrophotometer. The RNA was then reverse-transcribed into complementary DNA using the PrimeScript^TM^ RT reagent Kit with gDNA Eraser (Catalog No. RR037A, TaKaRa Bio Inc., Kusatsu, Japan). The reverse transcription reaction was performed under the following thermocycling conditions: 37 °C for 15 min, followed by enzyme inactivation at 85 °C for 5 s. Subsequently, quantitative Polymerase Chain Reaction was performed in triplicate using a SYBR Green detection system. Relative gene expression was calculated using the 2^−ΔΔCT^ method. Primer sequences are listed in Table S1 of the Supplementary Materials.

### 2.8. Metagenomic Sequencing and Bioinformatics Analysis

Total genomic DNA was extracted from the cecal contents, and shotgun metagenomic sequencing was performed on an Illumina platform. To ensure data reliability, the raw reads were subjected to rigorous quality control using fastp software (v0.23.4) to remove low-quality sequences and adapters. Host DNA contamination was subsequently filtered out by aligning the clean reads against the goose reference genome using Bowtie2 (v2.5.1). Following quality control, the high-quality reads were assembled into contigs using MEGAHIT (v1.2.9), and open reading frames were predicted using Prodigal (v2.6.3). A non-redundant gene catalog was then constructed using CD-HIT (v4.8.1) to perform clustering. For taxonomic and functional structural analysis, sequence alignment of the non-redundant gene set against the NCBI non-redundant protein database and the Kyoto Encyclopedia of Genes and Genomes (KEGG) database was performed using the Diamond tool (v0.8.35).

Taxonomic abundance and alpha diversity metrics (Chao1, Shannon, Simpson) were calculated using Mothur software (v1.48.0). Beta diversity was evaluated using Principal Coordinate Analysis (PCoA) based on Bray–Curtis distances. Community differences were assessed using analysis of similarities (ANOSIM) and linear discriminant analysis effect size (LEfSe, v1.1.2). The LEfSe analysis inherently controls for false discoveries by sequentially applying the non-parametric factorial Kruskal–Wallis sum-rank test, the pairwise Wilcoxon rank-sum test, and linear discriminant analysis (LDA) to estimate the effect size of each differentially abundant taxon. Taxa or pathways with an LDA score > 2.0 and *p* < 0.05 were considered significant biomarkers.

### 2.9. LC-MS Untargeted Metabolomics Analysis

Untargeted metabolomics profiling was conducted using liquid chromatography-mass spectrometry (LC-MS). Briefly, samples were thawed at 4 °C, and 100 μL aliquots were transferred to EP tubes, followed by addition of 400 μL acetonitrile-methanol (1:1). After vortexing (30 s), samples underwent low-temperature ultrasonication (5 °C, 40 kHz, 90 min) and were incubated at −20 °C for 30 min. Supernatants obtained after centrifugation (4 °C, 13,000× *g*, 15 min) were dried under nitrogen, reconstituted in 100 μL acetonitrile-water (1:1), ultrasonicated (5 min), and centrifuged (4 °C, 13,000× *g*, 5 min) for LC-MS analysis.

To ensure data reliability and monitor instrument stability, quality control (QC) samples were prepared by pooling equal aliquots from all experimental samples. During the LC-MS analytical run, a QC sample was injected continuously at the beginning of the sequence to equilibrate the chromatography column. Subsequently, a QC sample was injected at regular intervals (every 10 experimental samples) throughout the analytical batch to monitor instrument drift and assess overall batch stability. Data processing was performed using Progenesis QI (Waters Corporation, Milford, MA, USA). Metabolites were annotated by matching MS/MS spectra to Human Metabolome Database and Metlin Metabolite Database databases. Differential metabolites between pairwise comparisons were strictly defined using the following specific statistical criteria: a Variable Importance in Projection score > 1 derived from the OPLS-DA model, and a statistical significance of *p* < 0.05 calculated via a two-tailed Student’s *t*-test, with the *p*-values further adjusted for multiple testing using the Benjamini–Hochberg False Discovery Rate method.

### 2.10. Integrative Analysis of Metagenomic and Metabolomic Data

Differential taxa identified from metagenomics were integrated with differential metabolites from metabolomics. Spearman correlation analysis was performed using SPSS 27.0 (IBM Corp., Armonk, NY, USA) and Origin 2022 (OriginLab Corporation, Northampton, MA, USA). A strong microbe–metabolite correlation was defined as |r| > 0.6 with *p* < 0.05.

### 2.11. Data Analysis

As noted above, the individual goose served as the experimental unit for all statistical comparisons. Prior to any parametric statistical testing, all targeted physiological and phenotypic data were strictly evaluated to ensure they met the necessary statistical assumptions. Specifically, normal distribution of the data was confirmed using the Shapiro–Wilk test, and homogeneity of variances was verified using Levene’s test. Once these assumptions were validated, one-way analysis of variance was used to compare differences among the three dietary groups. When variances were homogeneous, the Least Significant Difference post hoc test was applied for multiple comparisons. *p* < 0.05 was considered statistically significant, and *p* < 0.01 highly significant. Results are presented as the mean ± standard error of means (SEM).

## 3. Results

### 3.1. Effects of Different Dietary Fiber Levels on the Intestinal Morphology of WWG

**Figure 1:**
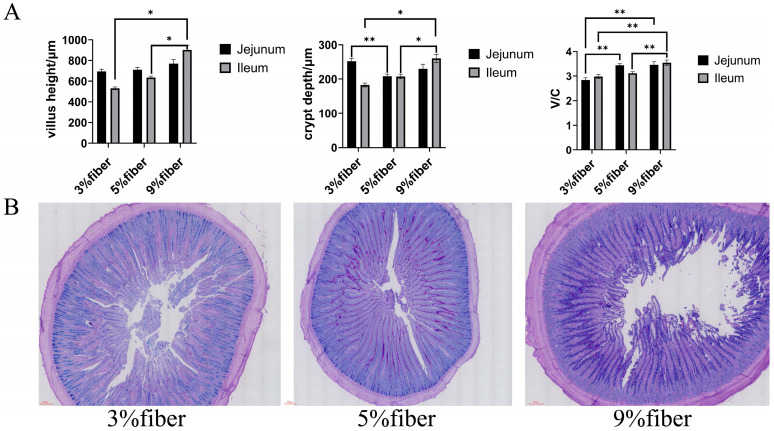
Effects of Different Dietary Fiber Levels on the Intestinal Morphology of WWG. (**A**) Effect of different fiber levels on the intestinal tissues of WWG. (**B**) Representative H&E stained micrographs of the jejunum and ileum from the 3%, 5%, and 9% fiber groups. Relevant histological structures are clearly labeled: V indicates villus, and C indicates crypt. Scale bars = 200 μm; Original magnification: 40×. As visually presented in the panels, increasing the dietary fiber level to 9% significantly enhanced the mucosal structural integrity, evidenced by visibly longer and denser villi compared to the lower fiber groups.* *p* < 0.05, ** *p* < 0.01.

As shown in Figure 1A. Jejunalcrypt in the 3% fiber group was significantly higher than in the 5% fiber group (*p* < 0.01), accompanied by a markedly lower villus-to-crypt ratio compared with both the 5% and 9% fiber groups (*p* < 0.01). In the ileum, both the 3% and 5% fiber groups exhibited significantly reduced villus height, crypt depth, and villus-to-crypt ratio relative to the 9% fiber group (*p* < 0.05 and *p* < 0.01).

As illustrated in Figure 1B, increasing dietary fiber levels improved jejunal VH and density, and fiber content exerted a pronounced influence on jejunal CD. Specifically, in the Jejunum, the CD in the 9% fiber group (770.30 ± 39.54 μm) was significantly higher compared to the 3% (695.71 ± 21.30 μm) and 5% (710.98 ± 21.91 μm) fiber groups (*p* < 0.01). The V/C ratio in the 5% (3.44 ± 0.07) and 9% (3.46 ± 0.12) fiber groups was significantly elevated relative to the 3% group (2.84 ± 0.09) (*p* < 0.01), whereas the CD in the 5% fiber group (208.18 ± 6.10 μm) was significantly reduced compared to the 3% group (251.98 ± 8.35 μm) (*p* < 0.05). In the ileum, the 9% fiber group exhibited significantly greater VH (903.78 ± 44.73 μm), CD (260.20 ± 12.31 μm), and V/C ratio (3.54 ± 0.11) compared to the 3% (VH: 531.44 ± 13.53 μm; CD: 182.78 ± 5.70 μm; V/C: 2.98 ± 0.08) and 5% (VH: 634.53 ± 13.81 μm; CD: 207.42 ± 6.33 μm; V/C: 3.12 ± 0.06) fiber groups (*p* < 0.01). Furthermore, histomorphological evaluation of the duodenum was also conducted; however, no significant differences in VH, CD, or the villus-to-crypt ratio were observed among the dietary treatments (Supplementary Table S2).

### 3.2. Effects of Different Dietary Fiber Levels on the Immune Function of WWG

**Figure 2:**
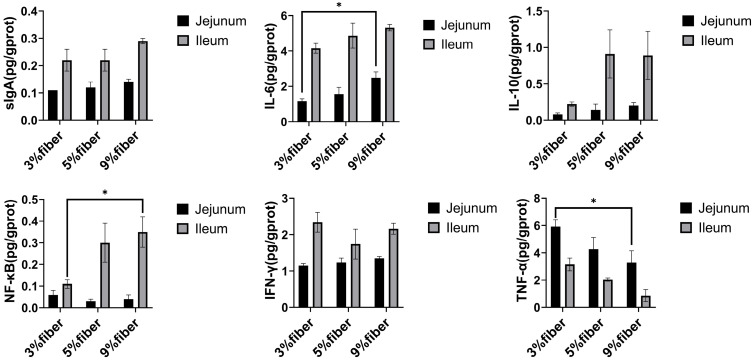
Effect of different fiber levels on intestinal immune function of WWG. * *p* < 0.05.

As shown in Figure 2, IL-6 levels in the jejunum were significantly lower in the 3% fiber group than in the 9% fiber group (*p* < 0.05), whereas TNF-α levels were significantly higher (*p* < 0.05). In the ileum, the 3% fiber group showed significantly reduced NF-κB levels compared with the 9% fiber group (*p* < 0.05), indicating a fiber-dependent modulation of local inflammatory responses.

### 3.3. Effects of Different Dietary Fiber Levels on Intestinal Gene Expression in WWG

**Figure 3:**
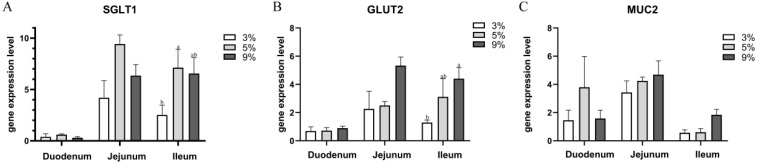
Effect of different fiber levels on the functional genes of the intestinal tract of WWG. (**A**) *SGLT1*; (**B**) *GLUT2*; (**C**) *MUC2*. Different lowercase letters (a, b, ab) indicate significant differences among groups (*p* < 0.05).

As depicted in Figure 3, ileal *SGLT1* expression was significantly regulated in the 5% fiber group compared with the 3% fiber group (*p* < 0.05), while *GLUT2* expression in the 9% fiber group was significantly higher than in the 3% fiber group (*p* < 0.05). No significant differences were detected in the expression levels of *SGLT1* and *GLUT2* in the duodenum or jejunum (*p* > 0.05). *MUC2* expression remained consistent across all dietary treatments and intestinal regions (*p* > 0.05).

### 3.4. Effects of Different Dietary Fiber Levels on the Microbial Diversity in the Intestinal Content of WWG

A total of 573,473 high-quality sequences were generated, corresponding to approximately 236.94 mega base pair of clean base data. Following de novo assembly and gene prediction, the non-redundant gene catalog was constructed. The average length of the predicted genes in this non-redundant catalog was 412.97 base pair (bp), ranging from 249 to 496 bp (Table S3 of the Supplementary Materials). These metrics confirm the reliability of the sequencing dataset for downstream microbial diversity and functional analyses.

**Figure 4:**
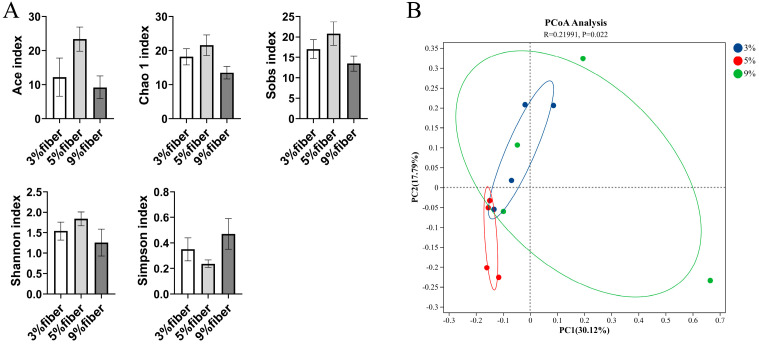
Effects of Different Dietary Fiber Levels on the Microbial Diversity in the Intestinal Content of WWG. (**A**) Alpha diversity between groups of WWG at different fiber levels. (**B**) Beta diversity analysis of cecum bacteria.

The alpha diversity of the gut microbiota was assessed using the Ace, Chao1, Sobs, Shannon, and Simpson indices. As shown in Figure 4A, the Ace and Chao1 indices exhibited marginal significance (*p* = 0.05) among the groups, while other indices such as Shannon and Simpson did not differ significantly (*p* > 0.05). This indicates that while dietary fiber levels had a limited impact on overall microbial evenness, they may have slightly modulated community richness. Furthermore, beta diversity analysis using PCoA based on Bray–Curtis distances (Figure 4B) revealed partial overlap among the treatment groups. However, to quantitatively evaluate the extent of structural divergence, ANOSIM based on Bray–Curtis distances matrix was conducted, which confirmed that the inter-group differences in overall microbial community structure were indeed statistically significant (R = 0.21991, *p* = 0.022).Therefore, while the lack of complete separation visually indicates that dietary fiber levels did not induce a wholesale restructuring of the overall microbial community, the robust ANOSIM results demonstrate a subtle yet statistically significant modulation of the cecal microbiota composition, primarily driven by shifts in specific taxa rather than divergent community-wide alterations.

**Figure 5:**
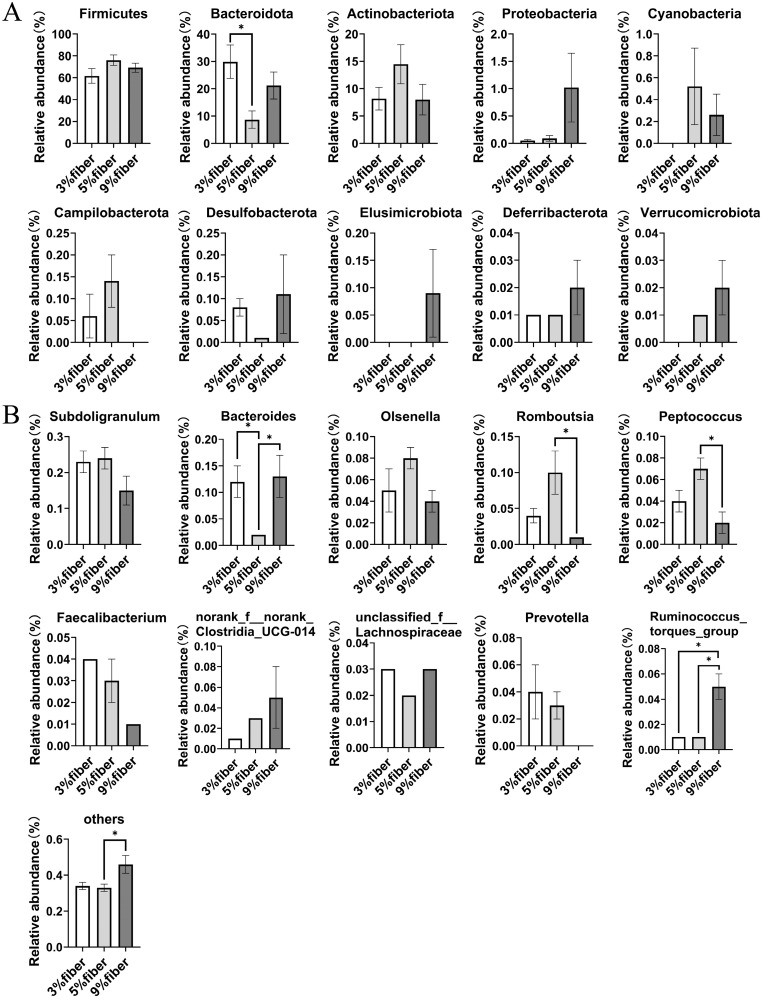
Comparison of the relative abundances of dominant gut microbiota among the different dietary fiber groups. (**A**) Group mean comparisons of the relative abundance of predominant bacteria at the phylum level. (**B**) Group mean comparisons of the relative abundance of predominant bacteria at the genus level. Data are presented as mean ± SEM. * *p* < 0.05.

As shown in the community bar plots (Figure S1A) and the intergroup comparison of mean abundances (Figure 5A), the predominant bacterial phyla included Firmicutes, Bacteroidetes, Actinobacteria, Campilobacterota, and Deferribacterota. Notably, the relative abundance of Bacteroidetes in the 3% fiber group was significantly higher than that in the 5% fiber group (*p* < 0.05). At the genus level (Figure S1B and Figure 5B), *Subdoligranulum*, *Bacteroides*, *Olsenella, Romboutsia*, *Peptococcus*, and *Faecalibacterium* were dominant. The relative abundance of *Bacteroides* was significantly higher in the 3% and 9% fiber groups than in the 5% fiber group (*p* < 0.05). *Romboutsia* abundance was significantly greater in the 5% fiber group compared with the 9% fiber group (*p* < 0.05), and *Peptococcus* exhibited a similar pattern (*p* < 0.05).

### 3.5. Microbial Species Differences in Intestinal Content of WWG Under Different Dietary Fiber Levels

**Figure 6:**
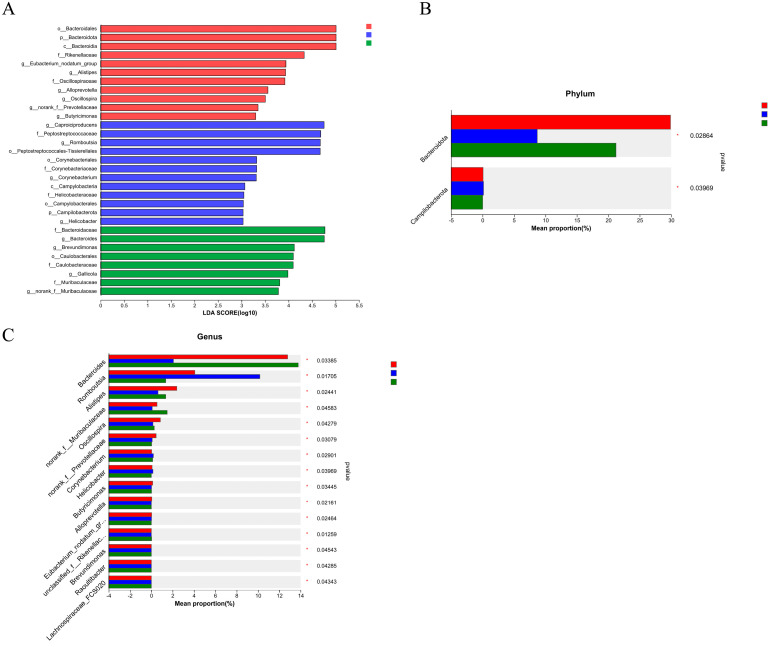
Microbial Species Differences in Intestinal Content of WWG under Different Dietary Fiber Levels. (**A**) LEfSe analysis of gut bacteria. (**B**) Analysis of species differences at the level of the cecum bacterial phylum. (**C**) Analysis of differential bacterial species at the genus level. Color legend: Red = 3% dietary fiber; Blue = 5% dietary fiber; Green = 9% dietary fiber. * denotes the *p*-value of inter-group difference, and *p* < 0.05 indicates a statistically significant difference.

As shown in Figure 6A, a total of 31 differential microbial biomarkers with a LDA score > 2.0 were identified. *Bacteroidota* was significantly enriched in the 3% fiber group and exhibited the highest LDA score. *Caproiciproducens* was significantly enriched in the 5% fiber group, whereas *Bacteroidaceae* was significantly enriched in the 9% fiber group (*p* < 0.05).

Figure 6B shows that two phyla exhibited significant variation among groups. *Bacteroidota* abundance in the 3% fiber group was significantly higher than in the 5% fiber group, while *Campilobacterota* abundance in the 5% fiber group was significantly higher than that in both the 3% and 9% fiber groups (*p* < 0.05).

Genus-level analysis (Figure 6C) identified *Bacteroides*, *Romboutsia*, *Alistipes*, and *norank_f__Muribaculaceae* as the primary taxa contributing to microbial differences among fiber levels.

### 3.6. Metabolomics Analysis

As illustrated in Figures S2 and S3 of the Supplementary Materials. Principal Component Analysis (PCA) and PLS-DA analyses revealed distinct clustering and clear separation of cecal metabolites among the 3%, 5%, and 9% fiber groups in both positive and negative modes. These results indicate substantial metabolic differences among the treatment groups. Permutation tests confirmed the robustness of the PLS-DA models, with Q^2^ intercepts below 0.05 in both ion modes, demonstrating the absence of overfitting.

**Figure 7:**
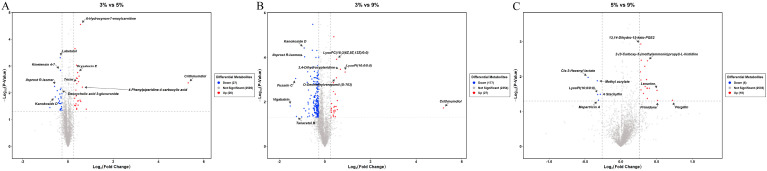
Differential metabolite volcano maps combining positive and negative ion modes. (**A**) Volcano map of differential metabolites between the 3% fiber group and the 5% fiber group; (**B**) Volcano map of differential metabolites between the 5% fiber group and the 9% fiber group; (**C**) Volcano map of differential metabolites between the 3% fiber group and the 9% fiber group. Red circles represent up-regulated differential metabolites, blue circles represent down-regulated differential metabolites, and grey circles represent metabolites with no significant change in both positive and negative ion modes.

As shown in Figure 7, comparisons of the 3% and 5% fiber groups revealed 137 metabolites in positive ion mode, of which 31 were differentially abundant (13 up-regulated and 18 down-regulated), and 199 metabolites in negative ion mode, with 22 being differentially abundant (13 up-regulated and 9 down-regulated). In the 3% vs. 9% comparison, 645 metabolites were detected in positive mode, with 137 differentially abundant (12 up-regulated and 125 down-regulated), and 588 in negative mode, with 67 differentially abundant (15 up-regulated and 52 down-regulated). For the 5% vs. 9% comparison, 65 metabolites were detected in positive mode, with 15 differentially abundant (12 up-regulated and 3 down-regulated), and 78 metabolites were detected in negative mode, with 8 differentially abundant (6 up-regulated and 2 down-regulated). To provide intuitive biological insights, the exact numbers of these altered metabolites, alongside the specific names of the top candidates exhibiting the most significant changes, have been explicitly annotated in the respective subplots of Figure 7.

**Figure 8:**
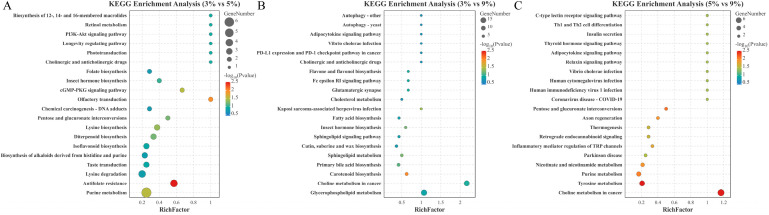
KEGG pathway enrichment analysis of differential metabolites in the cecal contents of WWG across different dietary fiber groups. (**A**) 3% vs. 5% fiber group. (**B**) 3% vs. 9% fiber group. (**C**) 5% vs. 9% fiber group. The *y*-axis represents the names of the enriched KEGG pathways, and the *x*-axis indicates the statistical significance (*p*-value). The size of each bubble corresponds to the number of differential metabolites annotated to a specific pathway, while the color gradient reflects the significance level.

KEGG enrichment analyses (Figure 8) revealed that in the 3% vs. 5% comparison, significantly enriched pathways included lysine biosynthesis, folate biosynthesis, and purine metabolism (*p* < 0.05). In the 3% vs. 9% comparison, significantly enriched pathways primarily involved glycerophospholipid metabolism, carotenoid biosynthesis, and primary bile acid biosynthesis (*p* < 0.05). For the 5% vs. 9% comparison, enriched pathways included amino acid metabolism, lipid, and carbohydrate metabolism, as well as riboflavin metabolism (*p* < 0.05).

### 3.7. Integrative Analysis of Gut Microbiota and Metabolomics

**Figure 9:**
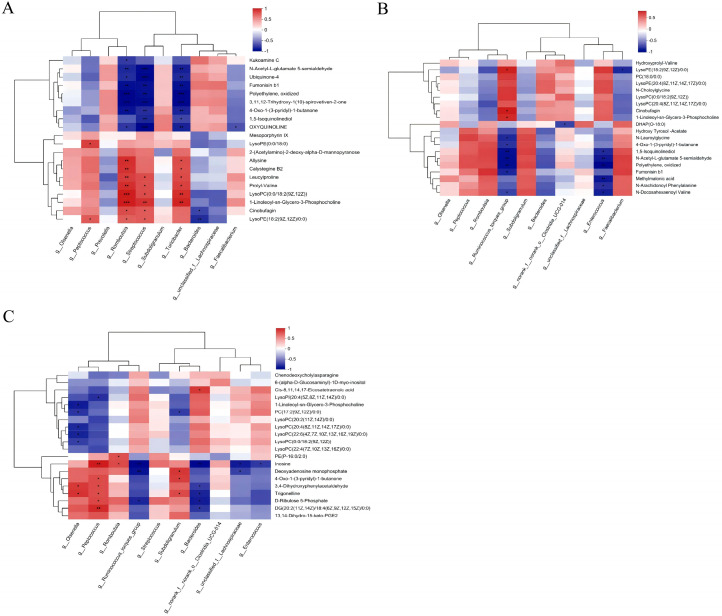
Spearman correlation heatmap between differential metabolites and gut microbiota at the genus level in the cecal contents of WWG across different dietary fiber groups. (**A**) 3% vs. 5% fiber group. (**B**) 3% vs. 9% fiber group. (**C**) 5% vs. 9% fiber group. The *x*-axis represents the relative abundance of microbial genera, and the *y*-axis represents the normalized intensity of the differential metabolites. Red grids indicate positive correlations, while blue grids indicate negative correlations. The intensity of the color corresponds to the magnitude of the Spearman correlation coefficient (r). Significant correlations are marked with asterisks: * *p* < 0.05, ** *p* < 0.01, *** *p* < 0.001.

To construct a coherent mechanistic framework linking dietary fiber to gut health, we prioritized the integrative analysis of significant microbe-metabolite correlations within three core biological pathways: lipid metabolism, amino acid (specifically lysine) biosynthesis, and purine metabolism (Figure 9). Alterations in lipid metabolism, which are essential for cellular membrane formation, were closely linked to specific microbial shifts. Notably, Peptococcus and the Ruminococcus_torques_group exhibited significant positive correlations with Lysophosphatidylethanolamine (LysoPE) and cinobufagin (*p* < 0.05). Conversely, Olsenella and Faecalibacterium showed significant negative correlations with specific lipid species, including 1-linoleoyl-sn-glycero-3-phosphocholine and LysoPE. This coordinated microbial modulation of the lipid pool may provide the necessary structural substrates for the rapid renewal of the intestinal epithelium observed in the high-fiber groups.

Driven by the profound impact of dietary fiber on immune parameters, we focused on amino acid biosynthesis, particularly lysine. Our network analysis revealed that Romboutsia and Turicibacter displayed highly significant positive correlations with lysine (*p* < 0.05), a critical building block for immune protein synthesis. Streptococcus also demonstrated positive associations with bioactive dipeptides such as leucyl-proline and prolyl-valine. These associations suggest that specific fiber-enriched taxa act as key contributors to the host’s amino acid pool, potentially supporting mucosal defense mechanisms.

Purine metabolism, which is strictly required for nucleic acid synthesis and energy transfer during rapid tissue development, was also significantly modulated. Subdoligranulum showed significant positive correlations with crucial purine intermediates, including inosine, deoxyadenosine monophosphate, and D-ribulose 5-phosphate. In contrast, genera such as Romboutsia, Peptococcus, and the Ruminococcus_torques_group exhibited negative correlations with these purine metabolites. These dynamic microbe-metabolite interactions highlight a complex regulatory network that balances cellular proliferation and energy supply within the developing gastrointestinal tract of brooding geese.

## 4. Discussion

Dietary fiber supplementation at appropriate levels can reshape the physiological structure and function of the poultry gastrointestinal tract, enhancing nutrient digestion and absorption, and ultimately promoting growth performance [13,14]. Conversely, insufficient fiber intake may induce intestinal atrophy, while excessive intake can cause mucosal abrasion and limit nutrient retention [15,16]. Therefore, determining the optimal dietary fiber levels for WWG is critical. Intestinal morphology is closely linked to absorptive capacity [17]. Furthermore, nutrient uptake relies heavily on intestinal transporters, primarily the glucose transporters *SGLT1* and *GLUT2* [18,19]. *SGLT1* is essential for the active transport of luminal glucose into enterocytes [20,21], while *GLUT2* further enhances glucose uptake and cellular transport [22]. In this study, the 9% fiber group exhibited significantly improved villus-to-crypt ratios in both the Jejunum and Ileum compared to the fiber-deficient 3% group. Concurrently, the expression of these key glucose transporters was upregulated in the higher fiber groups. These coordinated improvements suggest that increasing dietary fiber within an appropriate range structurally and functionally fortifies the intestinal absorptive surface during the early brooding period of WWG.

The intestine acts as a crucial immunological barrier protected by epithelial cells and tight junctions [23]. Activation of innate immunity releases pro-inflammatory cytokines (e.g., IL-6 and TNF-α), which can initiate inflammatory responses, disrupt tight junctions, and cause tissue damage [24,25], whereas IL-10 acts as an anti-inflammatory mediator to promote immune homeostasis [26,27]. NF-κB plays a central role in coordinating these responses [28,29,30], and dietary fiber is known to modulate this complex cytokine network [31,32]. Interestingly, our results revealed a differential modulation of intestinal cytokines by dietary fiber. While 9% fiber significantly decreased the potent pro-inflammatory cytokine TNF-α in the jejunum, it concurrently upregulated IL-6 and ileal NF-κB expressions compared to the 3% fiber group. Although IL-6 and NF-κB are traditionally recognized for their pro-inflammatory roles, their interpretation must be contextualized. In the current study, this immunological activation was accompanied by robust intestinal structural integrity (e.g., significantly increased villus height) without histological evidence of inflammatory damage. Therefore, we cautiously postulate that the moderate elevation of IL-6 and NF-κB may not indicate pathological inflammation, but rather reflect a state of physiological immune priming. During the rapid early development of brooding WWG, a basal level of innate immune signaling is often required to drive the maturation of gut-associated lymphoid tissues and to respond adaptively to the intense microbial fermentation stimulated by high dietary fiber. Nevertheless, the boundary between physiological immune maturation and inflammatory stress is subtle. The elevated expression of IL-6 and NF-κB could alternatively reflect a localized physiological stress response induced by the higher mechanical load of the 9% fiber diet. Future studies evaluating a broader panel of cytokines are required to fully elucidate this complex immune–microbiome–fiber crosstalk.

Gut microbial composition also plays a vital role in host metabolism. *Firmicutes* and *Bacteroidetes* are the dominant phyla, with *Firmicutes* enhancing energy harvest and *Bacteroidetes* contributing to nutrient absorption and ecological balance [33]. An increased abundance of *Firmicutes* is generally considered beneficial for host health because it enhances energy harvest, whereas a reduced abundance of *Bacteroidetes* may decrease the risk of pathogenic infections. In this study, *Firmicutes*, *Bacteroidetes*, *Actinobacteria*, and *Campilobacterota* were identified as the dominantbacterial phyla. At the genus level, the predominant genera included *Subdoligranulum*, *Bacteroides*, *Olsenella*, *Romboutsi-a*, *Peptococcus*, and *Faecalibacterium*. The phylum *Firmicutes* plays an important role in energy harvest from the diet in poultry, promoting caloric absorption and thereby supporting weight gain [34]. *Bacteroidetes* possess strong polysaccharide-degrading and fermentative capabilities, enabling the breakdown of dietary polysaccharides into metabolites such as short-chain fatty acids, which provide an additional energy source to the host [35]. The genus *Bacteroides* contribute substantially to poultry gut function by degrading non-digestible carbohydrates, alleviating inflammatory responses, promoting intestinal cell development, restoring mucosal vitality, and thereby inhibiting the proliferation of pathogenic bacteria [36]. Body fat deposition in animals has been linked to the relative abundance of *Firmicutes* and *Bacteroidetes* [37]. The genus *Romboutsia* primarily utilizes glucose, fructose, and maltose as sole carbon sources [38]. Previous studies have shownthat *Romboutsia* can effectively inhibit bacterial overgrowthin the duodenum, reduce bacterial translocation to distant organs, ameliorate pancreatitis pathology, and lower plasma levels of pro-inflammatory factors [39]. Furthermore, it can modulate gut microbiota-related metabolites and improve intestinal endothelial function in obese rats by regulating metabolic pathways such as glycerolipid metabolism, lipid catabolism, cholesterol metabolism, and insulin resistance [40]. In this study, the relative abundance of *Bacteroidetes* at the phylum level in the 3% fiber group was significantly higher than that in the 5% fiber group (*p* < 0.05). At the genus level, the relative abundance of Bacteroides in both the 3% and 9% fiber groups was significantly higher than that in the 5% fiber group, presenting an intriguing non-linear (U-shaped) response. Bacteroides species are renowned for their extensive repertoire of carbohydrate-active enzymes, granting them powerful polysaccharide-degrading capabilities. In the 9% high-fiber group, the abundant supply of complex dietary polysaccharides serves as an optimal substrate, potentially supporting their beneficial proliferation. Conversely, the elevated abundance of Bacteroides in the severely fiber-deficient 3% group highlights their remarkable metabolic plasticity. We hypothesize that when deprived of dietary glycans, certain Bacteroides species might shift their ecological niche to forage on host-secreted mucin glycoproteins for survival, as has been reported in other animal models [41,42]. While our study did not directly measure goblet cell activity or mucin degradation rates, and *MUC2* gene expression remained unaltered across groups (Figure 3C), such putative mucin foraging behavior could theoretically exacerbate mucosal vulnerability. If present, this potential mechanism may help explain why the 3% fiber group exhibited a compromised intestinal architecture (e.g., significantly reduced villus-to-crypt ratio) and altered local inflammatory profiles compared to the high-fiber groups. Conversely, the relative abundance of *Romboutsia* in the 5% fiber group was significantly higher than that in the 9% fiber group. Similarly, the relative abundance of *Peptococcus* in the 5% fiber group was significantly higher than that in the 9% fiber group (*p* < 0.05). These findings indicate that dietary fiber levels modulate the abundance of key bacterial groups, thereby influencing nutrient digestion and absorption efficiency, gut health, and ultimately the growth and development of WWG.

Metabolomics analysis revealed that different dietary fiber levels significantly altered the metabolite profiles in the intestinal content of WWG. PCA and PLS-DA analyses demonstrated distinct clustering and clear separation of intestinal metabolites among the different fiber groups in both positive and negative ion modes, indicating that dietary fiber levels induced substantial metabolic shifts. An exploratory analysis identified several putative differential metabolites between the groups, and these metabolites were enriched in several metabolic pathways. Specifically, in the 3% vs. 5% comparison, differential metabolites were significantly enriched in various biosynthesis pathways and purine metabolism. In the 3% vs. 9% comparison, significant enrichment was observed in biosynthesis pathways, nicotinate and nicotinamide metabolism, amino acid metabolism, and cholesterol metabolism. For the 5% vs. 9% comparison, the differential metabolites were mainlyenriched in amino acid metabolism, nicotinate and nicotinamide metabolism, lipid metabolism, purine metabolism, carbohydrate metabolism, and riboflavin metabolism. A paramount finding of this study, as highlighted in our title, is the profound impact of dietary fiber on microbial lysine biosynthesis. Lysine is widely recognized as the first or second limiting amino acid in poultry nutrition, playing an indispensable role in protein accretion and early developmental growth. Our metabolomics KEGG enrichment analysis revealed a significant upregulation of the lysine biosynthesis pathway when fiber levels were appropriately increased (e.g., in the 3% vs. 5% comparison). Furthermore, our integrative microbiome–metabolome analysis demonstrated that specific genera, notably Romboutsia and Turicibacter, appeared to exhibit strong positive correlations with intestinal lysine concentrations. This suggests a potential mechanism where specific dietary fiber levels enrich targeted microbial populations capable of de novo amino acid synthesis, which may theoretically contribute to the host’s nutritional pool [43,44]. However, direct in vivo validation using isotope tracing is required to definitively quantify this microbial contribution. Crucially, the biological significance of this microbial-derived lysine extends far beyond basic muscle growth; it is a vital metabolic cornerstone for immune homeostasis. Lysine and its downstream metabolites are strictly required for the rapid proliferation of intestinal immune cells (such as lymphocytes) and the active synthesis of functional immune proteins, including immunoglobulins (e.g., sIgA) and cytokines [45,46]. Therefore, we postulate that the association between fiber levels and enhanced microbial lysine biosynthesis may reflect a provision of essential biochemical substrates necessary to support the local immune maturation and mucosal defense mechanisms-such as the physiological upregulation of IL-6 and NF-KB-observed in our study. Changes in lipid metabolism affect energy storage and cell membrane formation, both of which are essential for maintaining cellular function. Purine metabolism is closely linked to nucleic acid synthesis and energy metabolism, and alterations in this pathway may influence cellular proliferation and energy supply. Collectively, these findings suggest that dietary fiber levels modulate multiple metabolic processes in the intestines of WWG, thereby influencing their growth performance.

Crucially, our integrative microbiome–metabolome analysis highlights significant associations between microbial fluctuations and host intestinal phenotypes. While specific metabolites identified in our network possess known biological implications for gut health in existing literature, it is important to note that our current multi-omics approach establishes correlations rather than direct causation. For instance, *Peptococcus* and the *Ruminococcus_torques_group* exhibited robust positive correlations with LysoPE. LysoPE is not only a fundamental structural component of cellular membranes but also a bioactive lipid molecule implicated in mucosal anti-inflammatory responses and barrier protection [47]. When contextualized with our morphological data—which demonstrated significantly increased villus heights and villus-to-crypt ratios in the 9% fiber group—the biological significance of this correlation becomes evident. The rapid renewal and massive proliferation of intestinal epithelial cells inherently demand a substantial supply of membrane lipids. We hypothesize that the dynamic interplay between dietary fiber—and potentially the varying dietary lipid sources—and taxa like *Peptococcus* is associated with the localized accumulation of LysoPE, thereby potentially acting as a crucial metabolic factor that supports rapid epithelial cell proliferation and fortifies the physical mucosal architecture [48]. Concurrently, the modulation of organic acids and structural derivatives by other key genera, such as *Romboutsia* and *Turicibacter*, further illustrates a synergistic microbial-metabolic network potentially linked to optimizing gut structural integrity and functional homeostasis [49]. Gut microbiota metabolize dietary fiber and other nutrients to produce diverse metabolites, which in turn influence microbial growth, proliferation, and functional activity. However, this study identified only correlations rather than causal relationships [50]. Specifically, the exact causal role of microbial lysine biosynthesis in mucosal immune maturation remains hypothetical. Further investigations are needed to elucidate the underlying mechanisms, which will provide a stronger theoretical basis for improving gut health in WWG through dietary fiber regulation. Despite the positive effects of the 9% fiber diet on intestinal morphometry, we acknowledge certain limitations in our study. Specifically, while improvements in villus-to-crypt ratios clearly indicate enhanced mucosal architecture and absorptive potential, these morphometric measurements alone are insufficient to definitively conclude an enhancement in overall intestinal barrier function. A comprehensive evaluation of barrier integrity ideally necessitates the assessment of epithelial continuity, goblet cell density, mucus layer quality, inflammatory infiltration, and the localization of tight-junction proteins (e.g., Claudin-1, Occludin, and ZO-1) [51]. Future studies incorporating these precise molecular and cellular parameters are warranted to fully elucidate the exact impact of dietary fiber on the physical and chemical barrier functions in brooding WWG. Furthermore, while the current study provides valuable insights into the microbiome–metabolome axis, the sample size used for high-throughput sequencing (*n* = 5 per treatment) represents another limitation. Although an *n* = 5 sample size is widely utilized in exploratory poultry omics research, it limits the overall statistical power and inherently increases the risk of false-positive associations within high-dimensional metagenomic and metabolomic datasets [52]. Consequently, the differential taxa and specific microbe–metabolite correlations identified in this study should be interpreted conservatively as preliminary or putative associations. Future large-scale cohort studies are necessary to rigorously validate these specific microbial shifts and metabolic biomarkers.

## 5. Conclusions

Appropriate dietary fiber supplementation improved intestinal morphology, enhanced antioxidant and immune function, and shifted microbial composition in WWG during the brooding period. While overall microbial diversity remained unchanged, fiber levels significantly influenced microbial abundance, particularly increasing *Bacteroidetes* and *Actinobacteria*, which are associated with fiber degradation and lipid metabolism. Fiber supplementation also altered cecal metabolite profiles, upregulating pathways related to amino acid, lipid, and purine metabolism, thereby supporting intestinal health. Integrated microbiome–metabolite analysis revealed that *Romboutsia*, *Streptococcus*, *Turicibacter*, *Ruminococcus_torques_group*, *Olsenella* and *Peptococcus* were significantly associated with multiple metabolites. Based on the combined morphological, immunological, microbial, and metabolic findings at day 28, the 9% dietary fiber level produced the most favorable outcomes for promoting the growth performance of WWG during the brooding period among the tested diets. While these results highlight the biological benefits of higher fiber inclusion, further studies incorporating a wider range of fiber gradients, multiple developmental stages, and different goose breeds are warranted to determine a universally optimal fiber requirement WWG.

## Figures and Tables

**Table 1:** Composition and nutrient levels of basal diets (air-dry basis) %.

Items	3% Fiber Group	5% Fiber Group	9% Fiber Group
Ingredients (%)			
Corn	53.78	45.51	25.35
Dehulled soybean meal	29	30	32.5
Rice hull powder	0	3.5	12.5
Wheat bran	14	15.5	18.5
Soybean oil	0	2.3	8
Limestone	0.75	0.75	0.72
caHPO4	1.4	1.37	1.33
NaCl	0.5	0.5	0.5
Premix 1	0.05	0.05	0.05
Vitamin premix 2	0.03	0.03	0.03
DL-Met	0.21	0.21	0.23
L-Thr	0.08	0.08	0.09
Choline chloride	0.2	0.2	0.2
Total	100	100	100
Nutrient levels 3			
CP/%	19.70	19.75	19.82
EE/%	3.05	2.86	2.39
ME/(MJ/kg)	11.81	11.75	11.59
CF/%	3.52	5.07	9.00
IDF/%	15.09	17.43	23.34
SDF/%	0.98	0.90	0.99
TDF/%	16.07	18.33	24.33
Ca/%	0.80	0.80	0.80
TP/%	0.77	0.76	0.75
AP/%	0.42	0.42	0.42
Lys/%	0.98	1.00	1.04
Met/%	0.50	0.50	0.50

(1) The mineral premix provided per kg of diet: Zn 90 mg, Mn 80 mg, Fe 80 mg, Cu 20 mg, I 0.35 mg. (2) The vitamin premix provided per kg of diet: VA 9000 IU, VD_3_ 3000 IU, VE 24 IU, VB_6_ 3 mg, VK_3_ 1.8 mg, VB_2_ 5 mg, VB_12_ 0.1 mg, niacin 40 mg, biotin 0.05 mg, D-pantothenic acid 15 mg. (3) Amino acids, crude fiber, insoluble dietary fiber, soluble dietary fiber, and total dietary fiber are measured values, all others are calculated.

## Data Availability

The raw data supporting the conclusions of this article will be made available by the authors on request.

## Supplementary Materials

The following supporting information can be downloaded at: https://www.mdpi.com/article/10.3390/ani16111709/s1, Table S1. Primer information for gut-associated genes in geese, Table S2. Effects of Different Dietary Fiber Levels on the Duodenal Morphology of WWG, Table S3. Metagenome sequencing results of intestinal microbial samples of WWG with different fiber levels, Figure S1. Microbial community composition profiles of the cecal contents in WWG across different dietary fiber groups, Figure S2. Total sample PCA analysis. Figure S3. PLS-DA analysis of metabolites of intestinal contents and substitution test.
