# Supplementary material for: Dietary Fiber Levels Modulate Intestinal Mucosal Architecture and the Microbiome–Metabolome Axis to Support Immune Homeostasis in Brooding Wanxi White Geese
Source: Animals (Basel). 2026 Jun 3;16(11):1709. doi: 10.3390/ani16111709 (PMC13255792; doi:10.3390/ani16111709)
Supplement: Supplementary file 1 [file animals-16-01709-s001.zip › animals-4269893-supplementary.pdf]

Table S1. Primer information for gut-associated genes in geese

Gene	Primer Sequences(5’-3’）	Tm（℃）
β-actin	F: GCCCAGCACGATGAAGAT<br>R: ATTTACGGTGGACGATGGAC	55.50
SGLT1	F: GTAACATTGGCAGCGGACAT<br>R: TGGGTACAAACAGCCATCCT	55.80
GLUT2	F: CAGTTCTTCCTGCTCCTGCT<br>R: TCATCGGGTCACAGTTTCCT	57.43
Mucin 2(MUC2)	F: ATGGGGCTAAGAATTGCCAGCCTCT<br>R: GACTTGCGATGTGGTT	55.10

Table S2. Effects of Different Dietary Fiber Levels on the Duodenal Morphology of Wanxi White Geese

**Items**	**3%**	**5%**	**9%**	***P*-Value**
villus height/μm	749.71±40.35	790.73±36.19	800.04±33.31	0.589
crypt depth/μm	227.02±17.81	219.20±11.04	229.85±12.28	0.857
V/C	3.63±0.12	3.83±0.15	3.67±0.19	0.617

Table S3. Metagenome sequencing results of intestinal microbial samples of Wanxi White Goose with different fiber levels

Samplelnfo	Seq_num	Base_num	Mean_length	Min_length	Max_length
XC1128	43,422	17,976,489	413.99496	280	496
XC1228	41,570	17,168,606	413.00472	337	478
XC1328	43,220	17,865,077	413.35208	262	448
XC1428	35,017	14,569,149	416.05931	312	489
XC1528	41,403	16,982,674	410.17979	262	491
XC2128	43,658	18,032,172	413.03248	320	431
XC2228	35,044	14,457,609	412.5559	341	442
XC2328	36,738	15,097,833	410.95958	363	430
XC2428	40,673	16,738,104	411.52863	363	481
XC2528	42,529	17,378,783	408.63371	250	430
XC3128	33,756	13,832,768	409.78694	364	430
XC3328	41,540	17,154,932	412.97381	249	431
XC3428	54,491	22,920,587	420.63069	364	455
XC3528	40,412	16,764,811	414.84735	262	430
Total	573,473	236,939,594	412.96713928	249	496

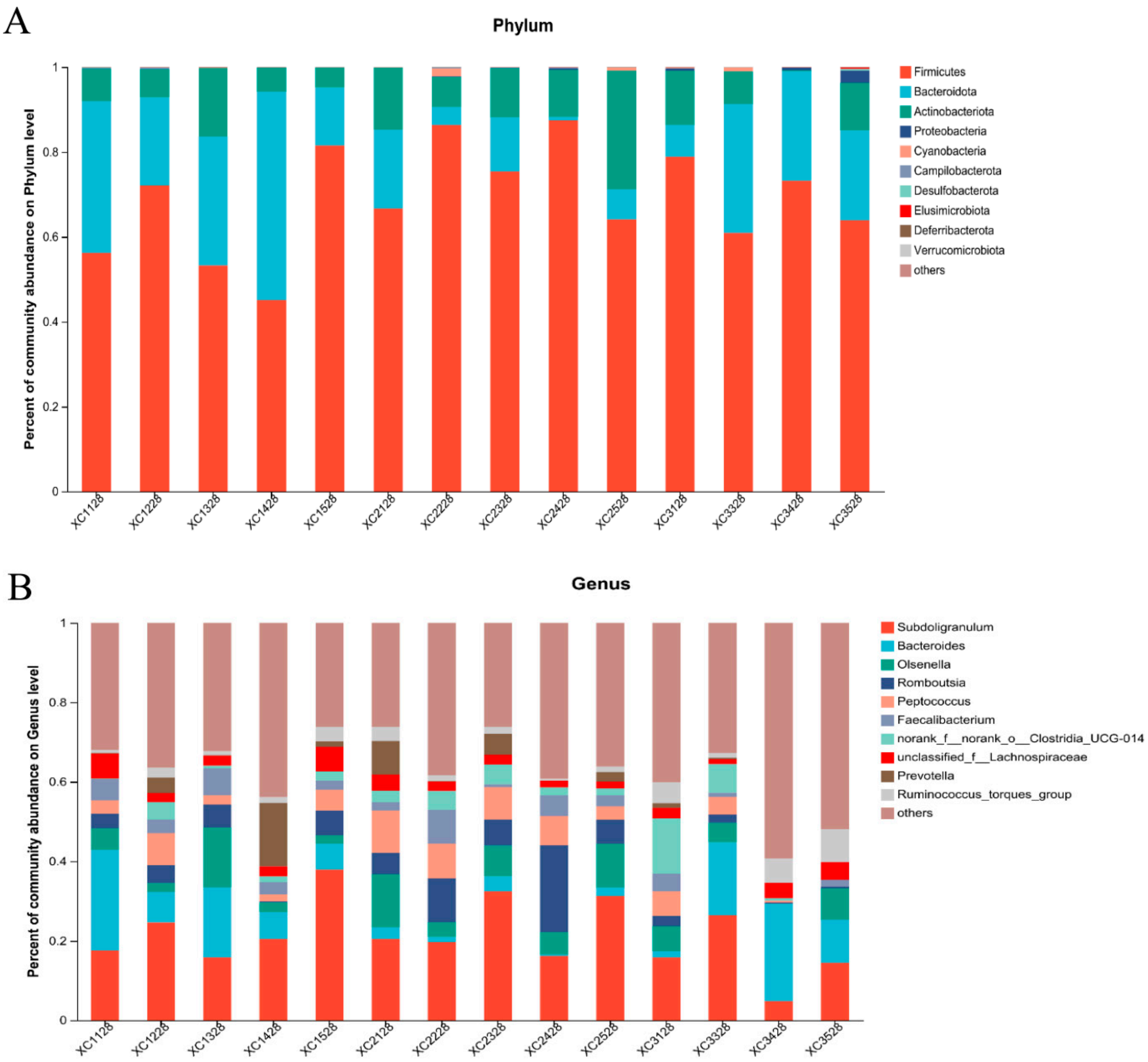

Fig. S1. Microbial community composition profiles of the cecal contents in WWG across different dietary fiber groups. (A) Relative abundance of bacterial taxa at the phylum level for each sample. (B) Relative abundance of bacterial taxa at the genus level for each sample.

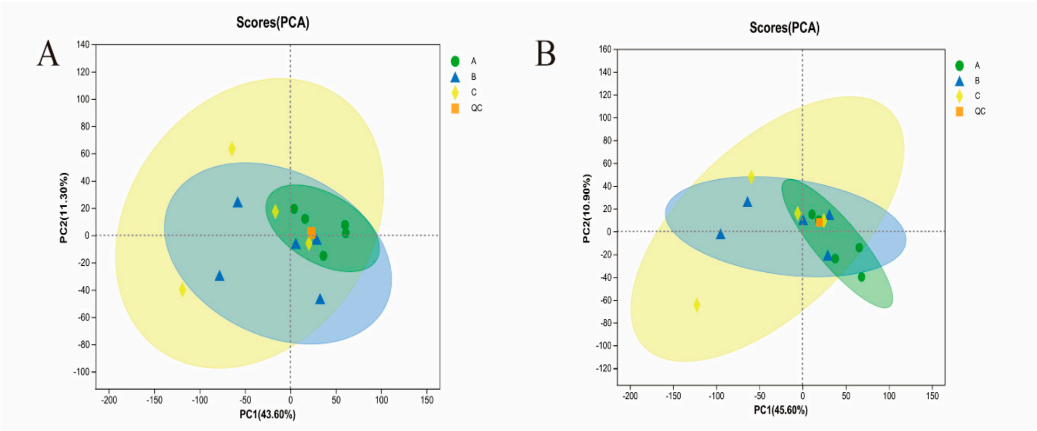

Fig. S2. Total sample PCA analysis. (A) PCA scores plot of positive ion metabolites. (B) PCA score plot of negative ion metabolites. PC1: scores of the first principal component; PC2: scores of the second principal component. Scatter point colors represent experimental groups, with the 95% confidence ellipse shown. Labels A, B, and C correspond to the 3%, 5%, and 9% fiber groups, respectively; the same convention applies hereafter.

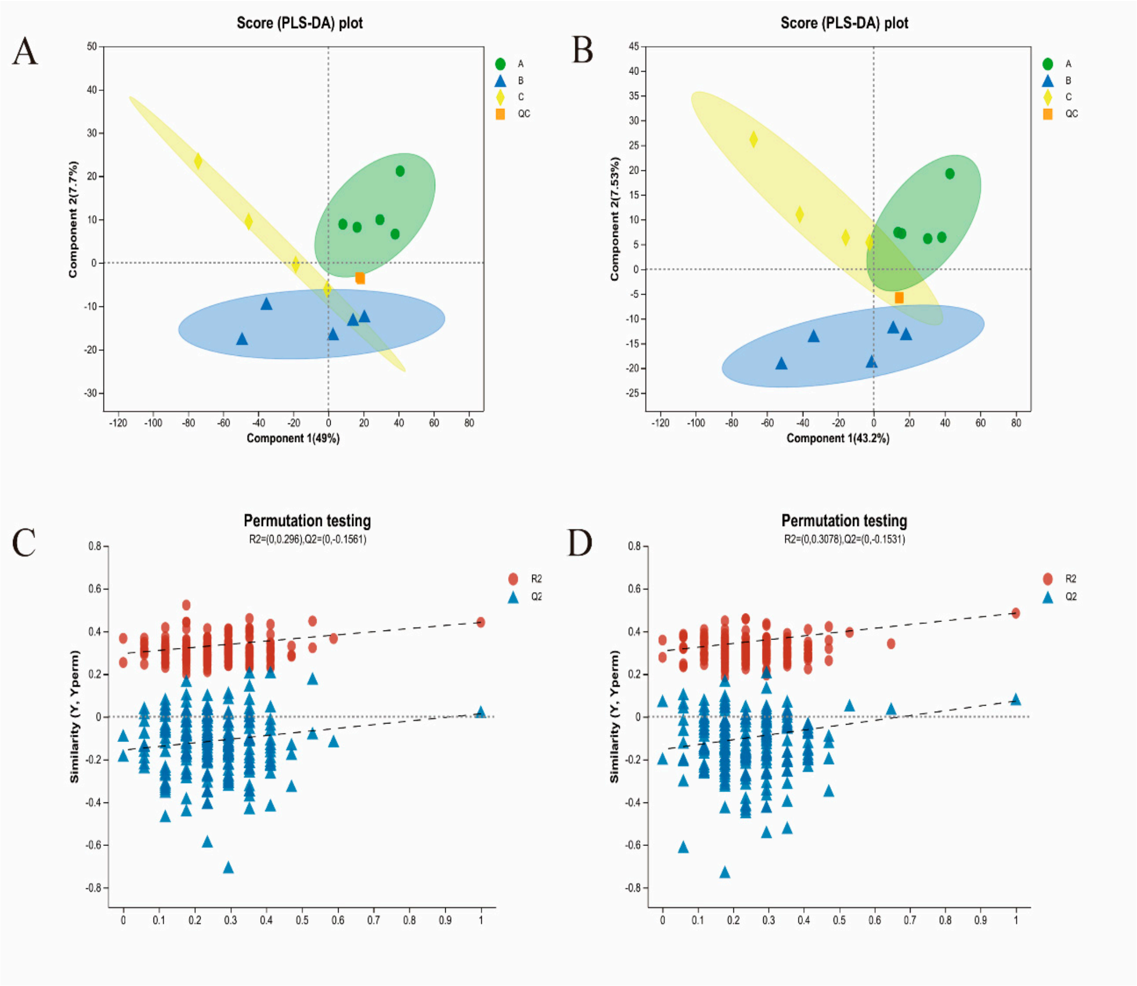

Fig. S3. PLS-DA analysis of metabolites of intestinal contents and substitution test. (A) PLS-DA score plot of intergroup samples in positive ion mode. (B) PLS-DA score plot of intergroup samples in negative ion mode. (C) Validation of the PLS-DA model for intergroup samples in positive ion mode. (D) Validation of the PLS-DA model for intergroup samples in negative ion mode.
